# The impact of the COVID-19 pandemic on nasopharyngeal carcinoma patients – a national cancer centre experience

**DOI:** 10.1007/s44178-023-00041-0

**Published:** 2023-06-07

**Authors:** Sharon S. Poh, Boon Fei Tan, Fang Yue Yong, Kam Weng Fong, Joseph T. S. Wee, Terence W. K. Tan, Melvin L. K. Chua, Kiattisa Sommat, Fu Qiang Wang, Yoke Lim Soong

**Affiliations:** 1 Division of Radiation Oncology, Duke-NUS Graduate Medical School, National Cancer Centre Singapore, Singapore, Singapore; 2 Duke-NUS Graduate Medical School, Oncology Academic Clinical Programme, Singapore, Singapore

**Keywords:** COVID-19, Nasopharyngeal carcinoma, Health services delivery

## Abstract

**Purpose or objective:**

The COVID-19 pandemic has resulted in significant healthcare implications, with care for cancer patients compromised due to resource diversion towards battling the pandemic. We aim to investigate the impact of the peak wave of the pandemic in 2020 on the delivery of cancer care in Singapore, specifically via our nasopharyngeal carcinoma (NPC) treatment data. This study applies real world numbers to the impact of COVID-19 on cancer care delivery in Singapore. The choice of nasopharyngeal cancer allows a good direct estimate of common treatment measures such as time to biopsy, time to staging scans, time to treatment commencement, due to its clear protocol and algorithms for staging and treatment; thus serving as an excellent surrogate for the effectiveness and timeliness of the different aspects of cancer care delivery.

**Materials and methods:**

In this retrospective study, we included all patients with newly diagnosed NPC from 1st January to 31st May from 2017 to 2020 at our centre. This time period was chosen as it coincided with the period in 2020 during the COVID-19 pandemic where there was the most strain on healthcare resources and the most restrictions on population movement within Singapore, which may impact on healthcare seeking behaviour. Narrowing down the time period to the first 5 months of the 4 respective years also allowed us to reduce the effect of annual seasonal variation in patient numbers seen as a result of holidays and festive periods such as the Lunar New Year and scheduled school holidays. Electronic medical records (EMR) were accessed. Only newly diagnosed NPC cases were included in our analysis. Patients with second synchronous primary malignancies or NPC disease recurrence were excluded. Data analysis was carried out using a combination of SPSS and Microsoft Excel.

**Results:**

Significantly, there was a reduction of 37–46.3% in newly diagnosed NPC cases during the peak of the COVID-19 pandemic from January to end May 2020 compared to the preceding three years. Despite the reduction in numbers of newly diagnosed NPC, there was no statistically significant differences in delay from biopsy to the first radiation oncology visit and from biopsy to the first day of treatment in 2020 compared to the preceding years. All the patients treated in our centre also received the standard NPC treatment for their disease stage as per international guidelines.

**Conclusion:**

We recommend a heightened awareness of the dangers of delaying cancer presentation and care in healthcare policies and resource allocation and at the same time, encourage patient’s confidence in their ability to seek care. With the resurgence of new COVID-19 variants and case numbers worldwide and in Singapore, this study focuses upon the need to be aware of the exigencies of other clinical groups in resource utilization. It would be instructive to compare this study with future long term follow up to investigate the trajectory of our cancer care delivery, as well as survival outcomes.

## Introduction

The COVID-19 pandemic has resulted in changes worldwide with significant socio-economic and healthcare implications. During this exceptional time, the care for cancer patients has been compromised due to diversion of healthcare resources towards battling the pandemic, with an anticipation of increased cancer mortality rate in the coming years [1]. The timely diagnosis and management of cancer is paramount. On the other hand, as cancer patients are at higher risk of complications and worse outcomes from COVID-19 infection [2], ensuring prompt cancer treatment whilst limiting their exposure to COVID-19 remains a challenge.

Worldwide, there are many layers in cancer care that have been affected, from screening, biopsies, laboratory analysis, imaging, surgery, chemotherapy and radiotherapy; not just the actual service but also the supply chain, technical maintenance and patient access [3]. City lockdowns, movement restrictions, increased burden of primary and secondary care in managing COVID-19 patients and suspected cases, and internalized fear of the general population to leave the safety of their homes contribute to reduced medical seeking behaviour with resulting delayed diagnosis, higher tumour staging and possible poor survival outcomes. Even with timely presentation, the prioritization of healthcare resources in containing and treating COVID-19 patients means that the delay in providing time-sensitive and life-saving cancer treatment was inevitable [4-6]. We may foresee higher cancer stages and an overall increase in cancer mortality in the years to come as a result of COVID-19.

**Fig. 1:**
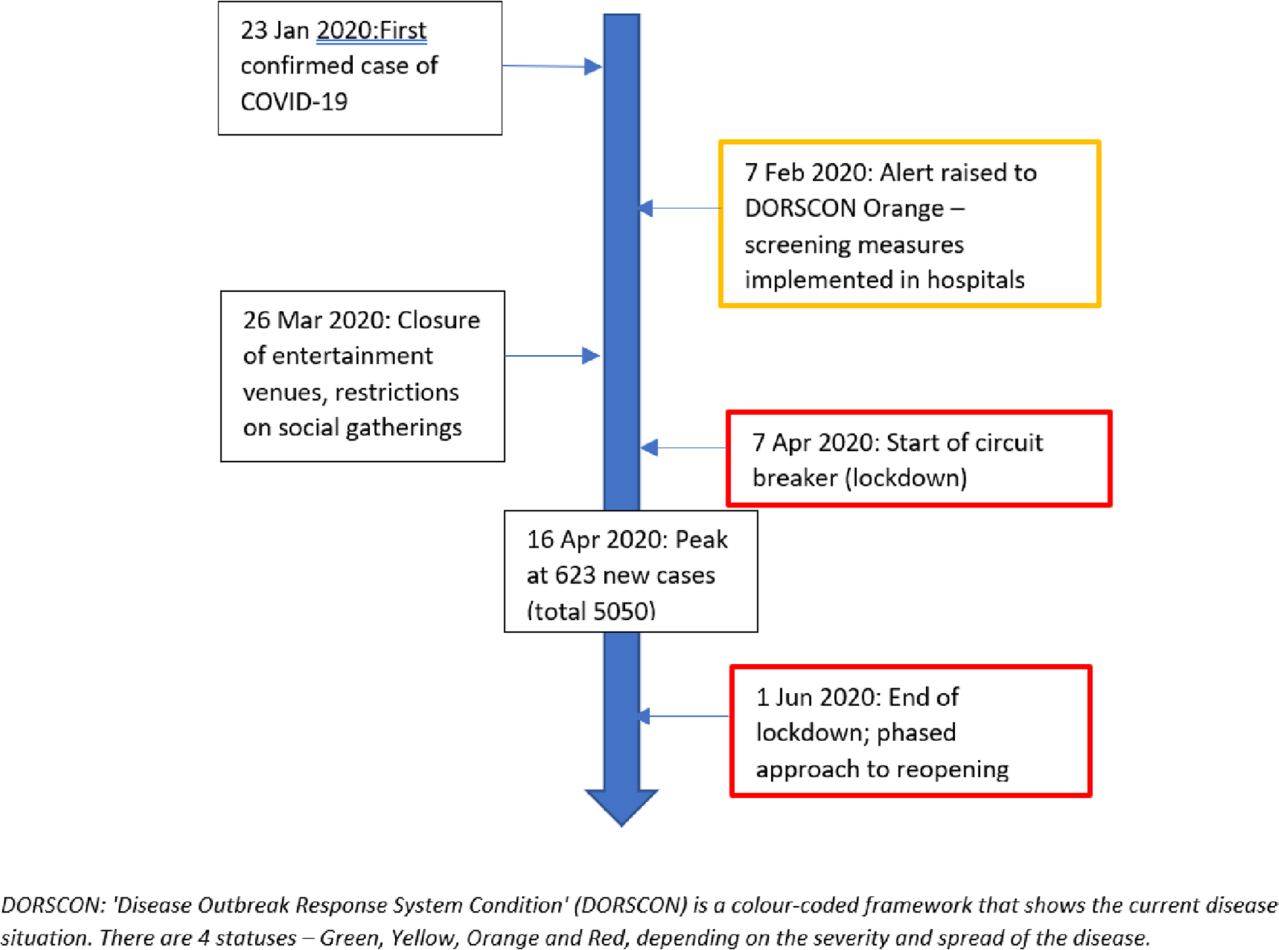
Timeline of Singapore’s COVID-19 situation and response measures

In Singapore, the first few imported cases of COVID-19 were detected in mid to late January 2020, with the country subsequently gradually raising the alert level from early February to March 2020, along with advice and restrictions to stay home and avoid going out unless for essential tasks and errands. This culminated in Singapore going into a lockdown from 7^th^ April to 1^st^ June 2021 over a period of 8 weeks at the peak of COVID-19 infection to curb transmission of unlinked infections (Fig. 1). The total number of COVID-19 infections during that period was about 45,000, with foreign workers living in dormitories forming the main affected population [3]. As a result, for the most part of the first half of 2020, the country’s healthcare system has had to pre-emptively restructure its services to cope with the burden of the pandemic by limiting unnecessary hospital visits, reducing non-essential services and focusing manpower and resources to managing COVID-19-related issues. In addition to this, stringent checks on travel history, body temperature, as well as the presence of any possible COVID-19 symptoms were instituted at all healthcare institutions in order to reduce the risk of healthcare – associated transmissions, which may serve to further impact the medical seeking behaviour of patients.

In this paper, we aim to investigate the impact of the COVID-19 pandemic on cancer care in our institution via a review of our nasopharyngeal carcinoma (NPC) case numbers. We specifically decided to look at nasopharyngeal carcinoma as a bellwether of our management of other cancer conditions in our centre during the pandemic, for the following reasons:

- 1. The management of this cancer has a clear standardized algorithm of management from diagnosis to treatment in our centre,
- 2. The majority of the treatment is managed and coordinated by a single provider (the radiation oncologist),
- 3. There is the ability to identify clear and consistent care delivery time points,
- 4. Our institution has historically treated the highest proportion of nasopharyngeal carcinoma patients nationally.

Singapore has one of the highest rates of NPC in the world at 6.7 per 100,000 [7]. In Singaporean males, nasopharyngeal cancer is in the top 10 most frequently occurring cancer in those aged 30–50 years old and the 8th most frequent cancer death [8]. Patients with suspected NPC are usually referred to Ear, Nose and Throat (ENT) specialists from primary care physicians. In the ENT clinic, a biopsy is done of the suspicious nasopharyngeal lesion and if this confirms NPC, the patients are referred on to the Radiation Oncology department in our centre. Here, we are responsible for the coordination of staging scans as well as treatment of the disease with radiation therapy with or without induction or concurrent chemotherapy. Thus, the workflow for NPC involves minimal external influences that may skew the measured timings relevant to this study, providing a clear picture of the impact of COVID-19 on any diagnostic and treatment timing delays. We compare this with data from pre-COVID years to ascertain if any trend was associated with the pandemic.

## Methods

In this retrospective study, we included all patients with newly diagnosed nasopharyngeal cancer from 1^st^ January to 31^st^ May from 2017 to 2020 at the National Cancer Centre Singapore. This time period was chosen as it coincided with the period in 2020 during the COVID-19 pandemic where there was the most strain on healthcare resources and the most restrictions on population movement within Singapore, which may impact on healthcare seeking behaviour. Narrowing down the time period to the first 5 months of the 4 respective years also allowed us to reduce the effect of annual seasonal variation in patient numbers seen as a result of holidays and festive periods such as the Lunar New Year and scheduled school holidays.

**Fig. 2:**
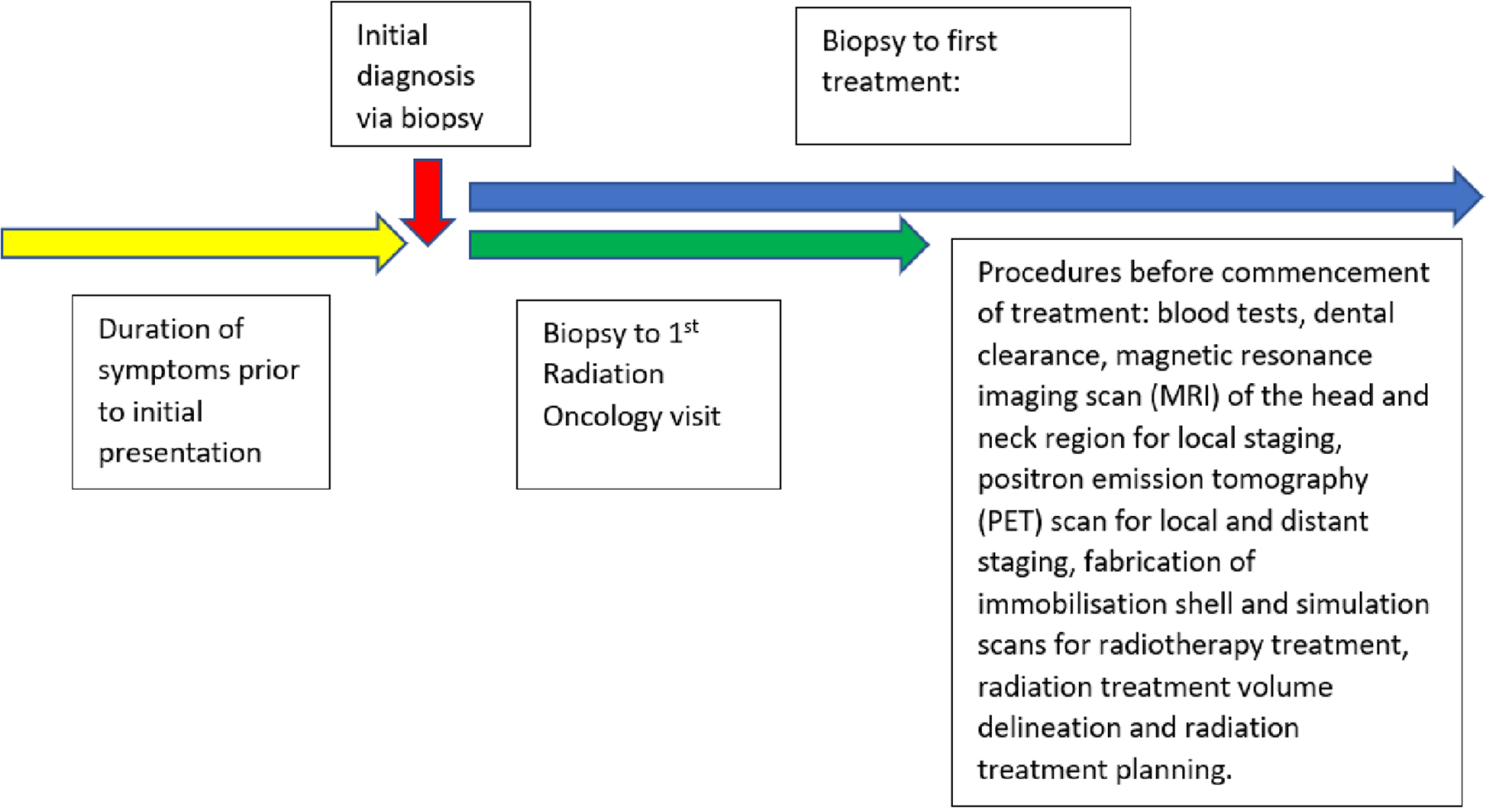
Data collection timepoints

Electronic medical records (EMR) were accessed to obtain information on patient demographics, disease information, pathology findings, as well as treatment details. Only newly diagnosed NPC cases were included in our analysis. Patients with second synchronous primary malignancies or NPC disease recurrence were excluded. Data was extracted based on the stated timepoints (Fig. 2). Within the timepoint from first visit to the radiation oncologist to the commencement of treatment, the following procedures are involved: blood tests, dental clearance, magnetic resonance imaging scan (MRI) of the head and neck region for local staging, positron emission tomography (PET) scan for local and distant staging, fabrication of immobilisation shell and simulation scans for radiotherapy treatment, radiation treatment volume delineation and radiation treatment planning.

Patients included in this study were all staged according to the American Joint Community on Cancer (AJCC) 8^th^ Edition. They were all treated with intensity modulated (IMRT) or volumetric arc (VMAT) technique radiation therapy. Registration of diagnostic MRI with planning computed tomography (CT) images was performed for all patients to aid delineation of target volumes and critical structures. The dose to gross disease with margin was 69.96 Gy in 33 fractions in 2.12-Gy fractions, whereas the rest of the volume received 60 Gy in 1.82-Gy fractions over the same period. We continued treatment of our patients with standard RT treatment doses and regiments, and did not use shortened or hypofractionated radiation treatment regiments for any of our patients during the COVID period. Statistical analysis was carried out utilizing a combination of Microsoft Excel and SPSS (IBM).

## Results

**Fig. 3:**
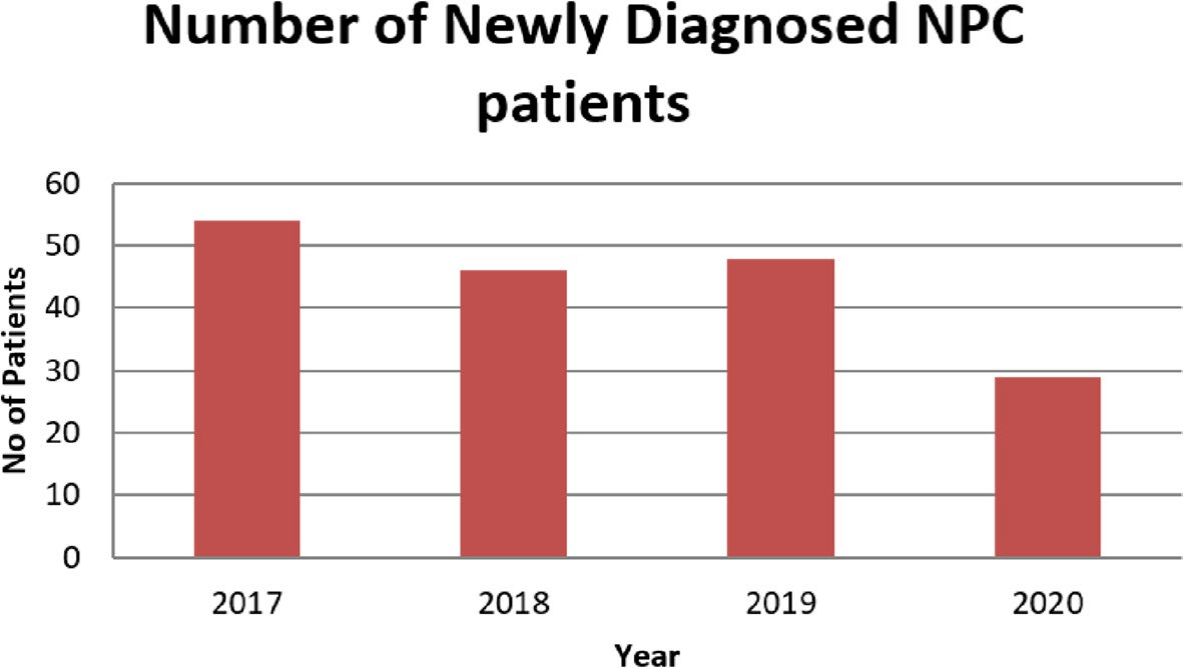
Number of newly diagnosed NPC patients in the month of January to 31^st^ May for the years 2017 to 2020

A total of 177 patients were newly diagnosed with nasopharyngeal cancer in our institution from 2017 to 2020 in the months of January to end of May. There were 54, 46, 48 and 29 patients in the years 2017, 2018, 2019 and 2020 respectively (Fig. 3). Missing data was attributable to several factors; namely patients who presented for first consultation with the radiation oncologist post diagnosis but failed to complete their staging investigations, incomplete record keeping especially for those who were referred by clinics or centres not within the public healthcare system, as well as data that could not be accessed due to lack of or withdrawal of patients’ consent.

There was no statistically significant difference in the age demographics of the patients, with the largest number of patients coming from the 51 to 70 years age group in all the 4 years, as per the natural history of NPC. The COVID-19 pandemic peak did not appear to alter the health seeking behaviour of the patients across the age groups. More male patients than female presented in 2020 (89.7% vs 10.3%), compared with that of previous years.

Table 1 shows the NPC disease stages at presentation for each year, as well as the data for the time points of interest, namely:

- i) the duration of symptoms prior to initial presentation. Most, if not all NPC patients will get their biopsy performed during the first visit at initial presentation, as the diagnostic method is via post-nasal space biopsy, which is performed as an in-clinic procedure.
- ii) the days from biopsy to the patient’s first Radiation Oncology visit in our centre.
- iii) the duration from biopsy to the first day of treatment.

**Table 1:** Disease and treatment characteristics

	2017 (*n* = 54)		2018 (*n* = 46)		2019 (*n* = 48)		2020 (*n* = 29)	
**Age (years)**								
≤ 30	2 (3.7%)		0 (0%)		0 (0%)		2 (6.9%)	
31—50	18 (33.3%)		19 (41.3%)		11 (22.9%)		5 (17.2%)	
51 – 70	29 (53.7%)		25 (54.4%)		32 (66.7)		15 (51.7%)	
≥ 71	5 (9.3%)		2 (4.3%)		5 (10.4%)		7 (24.1%)	
**Gender**								
Male	43 (79.6%)		30 (65.2%)		36 (75.0%)		26 (89.7%)	
Female	10 (20.4%)		16 (34.8%)		12 (25.0%)		3 (10.3%)	
**Stage**								
tage 1	2 (3.7%)		5 (10.9%)		2 (4.2%)		3 (10.3%)	
Stage 2	11 (20.4%)		9 (19.6%)		10 (20.8%)		7 (24.1%)	
Stage 3	20 (37.0%)		15 (32.6%)		20 (41.7%)		9 (31.0%)	
Stage 4A	19 (35.2%)		9 (19.6%)		14 (29.2%)		10 (34.5%)	
Stage 3&4A	39 (72.2%)		24 (52.2%)		34 (70.8%)		19 (65.5%)	
Missing	0 (0%)		2 (4.2%)		8 (17.4%)		2 (3.7%)	
**Duration of symptoms prior to initial presentation**								
< 30 days	6 (11.1%)		18 (39.1%)		8 (16.7%)		8 (27.6%)	
30-90 days	24 (44.4%)		19 (41.3%)		13 (27.1%)		15 (51.7%)	
3-6 months	11 (20.4%)		6 (13.0%)		16 (33.3%)		2 (6.9%)	
> 6 months	9 (16.7%)		3 (6.5%)		9 (18.8%)		4 (13.8%)	
Within 90 days	30 (55.6%)		37 (80.4%)		21 (43.8%)		23 (79.3%)	
Missing	0 (0%)		2 (4.2%)		0 (0%)		4 (7.4%)	
**Days from biopsy to 1**^**st**^** Radiation Oncology visit (days)**								
Median	10		12		14		13	
**1**^**st**^** treatment**								
Induction chemo	1 (1.9%)		1 (2.2%)		7 (14.6%)		14 (48.3%)	
CCRT	36 (66.7%)		30 (65.2%)		29 (60.4%)		6 (20.7%)	
RT alone	17 (31.5%)		14 (30.4%)		12 (25.0%)		9 (31.0%)	
Missing	0 (0%)		0 (0%)		1 (2.2%)		0 (0%)	
**Duration from biopsy to D1 treatment (days)**								
Median	33		35		40		33	
**Duration from biopsy to D1 of specific treatment (median days)**								
Induction chemo	NA		NA		14		29	
CCRT	33.5		35		39		37.5	
RT alone	32		38		40		35	
**3 year survival outcomes**								
Local recurrence (LR)	13 (24.1%)		4 (8.7%)		6 (12.5%)		6 (20.6%)	
	**Stage**		**Stage**		**Stage**		**Stage**	
	I	0	I	0	I	0	I	1
	II	3	II	1	II	0	II	1
	III	3	III	0	III	4	III	2
	IVA/B	7	IVA/B	3	IVA/B	2	IVA/B	2
Distant metastasis (DM)	11 (20.4%)		5 (10.9%)		5 (10.4%)		5 (17.2%)	
	**Stage**		**Stage**		**Stage**		**Stage**	
	I	0	I	0	I	0	I	1
	II	1	II	1	II	0	II	0
	III	5	III	0	III	1	III	2
	IVA/B	5	IVA/B	4	IVA/B	4	IVA/B	2
LR and DM	7		1		0		3	
Deaths	11		6		6		5	

Abbreviations: *CCRT* Concurrent chemo-radiation, *RT* Radiation therapy

Radical treatment for NPC generally falls into 3 regimens;

- 1. radiation therapy (RT) alone, which is generally used in Stage I and early Stage II disease, or in patients unfit to receive any chemotherapy (AJCC 8^th^ edition).
- 2. concurrent chemoradiation therapy, which is generally used in some Stage II and selected Stage III disease.
- 3. induction chemotherapy for 3 cycles, then concurrent chemoradiation therapy for more advanced disease or concurrent chemoradiation followed by adjuvant chemotherapy.

We analysed the data for each treatment regimen separately due to the different treatment planning duration needed for each regimen. For example, radiation therapy treatment planning in our centre takes an average of 10 to 14 days of planning time, which is not required in cases where the first treatment the patient receives is induction chemotherapy.

Another caveat in analyzing the data would be that the induction chemotherapy followed by concurrent chemoradiation treatment for advanced NPC (Stage III and up, excluding cT3N0M0 disease) only gained traction in mid to late 2019 after positive Phase III trial data [9], thus most of these patients received concurrent chemoradiation in the prior years.

There was a reduction of 37–46.3% in newly diagnosed NPC during the peak of the COVID-19 pandemic months of January to June 2020 (*n* = 29) compared to the same months in the preceding three years. However, no trend in stage migration to higher numbers of Stage III and IV disease was observed, with a comparable percentage of patients presenting consistently for each stage of disease.

We also did not observe any delay in presentation during the COVID-19 peak months in 2020 compared with previous years, with the majority (79.3%) of patients presenting within 90 days from development of initial symptoms.

Importantly, there was no delay in delivery of service by our centre during the peak COVID-19 pandemic period. We were able to keep consistently to seeing the patient within 2 weeks of biopsy (median = 13 days), which is similar to our median data across previous years. Also, for patients who received a radiation – based regimen (radiation therapy alone or concurrent chemo-radiation), there was no change in the median number of days taken from biopsy to first day of treatment, with a median of 35 to 37.5 days.

A comparison of the 3 – year survival data for each of the cohorts was also carried out, with the cut-off for analysis of the 2017 cohort at end – 2019, 2018 at end – 2020, 2019 at end – 2021 and 2020 at end – 2022. While the overall sample size was small, thus limiting statistical inference, both 3 – year local recurrence (LR) and distant metastasis (DM) rates appear to be higher than previous cohorts in 2018 and 2019, at 20.6% and 17.2% respectively. In addition, there were 2 patients with early—stage NPC who recurred within 3 years. 1 patient with Stage I disease developed both LR and DM, while another with Stage II disease developed LR. Both patients did not delay from development of initial symptoms to presentation at the ENT clinic; however, both patients did have a delay from biopsy to first Radiation Oncology clinic visit (27 days and 41 days; median 13 days), as well as a resulting delay from biopsy to commencement of treatment (59 days and 78 days; median 33 days). A deeper multi – centre and possibly international study would be of interest to further investigate this trend.

## Discussion

In this study, we provide a first look into the impact of COVID-19 on the presentation and management of NPC in a national cancer centre.

Significantly, there was a reduction of 37–46.3% in newly diagnosed NPC cases during the peak of the COVID-19 pandemic months of January to end May 2020 compared to the same months in the preceding three years. Whilst there are no similar published studies looking into NPC, studies in Italy, France and England similarly showed a reduction in cancer diagnosis during the peak of COVID-19 pandemic, ranging from 6.8–44.9% [10-12]. We postulate that this may be attributed to the following reasons:

- i) delay in presentation. As early presentations of NPC often show minor symptoms that are easily brushed aside, such as blocked nose, blocked ear, blood streaks in the sputum etc., patients may opt to delay seeking treatment during this period due to preoccupation with the pandemic and its accompanying socioeconomic complications, as well as a reluctance to visit healthcare institutions due to a perceived fear of infection, as well as inconvenience due to the stepped up screening measures instituted.
- ii) delay in diagnosis. During the study period of January to June 2020, and especially during the height of the pandemic from April to June in Singapore, healthcare resources were diverted to management of the increasing number of COVID-19 cases, with diversion of manpower and healthcare facilities to the COVID-19 efforts. Across public and private healthcare institutions, supposed non-urgent cases were also postponed or delayed. While cancer diagnosis is of course classified as urgent, it must also be noted that many NPC cases are picked up incidentally from other complaints such as blocked ear with gradually worsening hearing loss, or blocked nose, due to its insidious presentation, which may have received lower priority triaging during this period.
- iii) reduction in foreign patient numbers. In our centre, we do get a number of foreign patients from neighbouring countries. Due to the border closures, this source of patients has been eliminated. Interestingly however, we note that there was no stage migration to higher stages of disease at presentation. We postulate that this is due to the fact that NPC generally progresses at a moderate rate of progression and takes on average 6 to 12 months to develop and become symptomatic. Also, at more advanced stages of disease, the symptoms tend to become more significant and worrying, thus patients are more likely to seek treatment then.

In a French study, amongst the different malignancies investigated, head and neck cancers had lower rates of delay in diagnosis (-7.4%). The greatest decrease was seen in prostate, breast, bladder and colorectal cancer [1, 4]. The delay in prostate cancer diagnosis could be attributed to clinical triaging between the low and high risk groups, in which treatment in the low risk group can be delayed as cancer mortality rate is deemed not to be high. Meanwhile, the decrease in colorectal and breast cancers could be attributed to reduced national screening services to allow the appropriation of healthcare resources to COVID-19. In NPC, there is no screening program. Patients may present with upper respiratory symptoms such as blocked nose and rhinorrhoea. On one hand, these patients may self-isolate due to concerns of being infectious. On the other hand, these patients may be more likely to seek medical review to rule out COVID-19. Interestingly, our cohort of patients presented earlier (within 90 days of symptom onset) (79.3%) compared to that in 2017 and 2019, which may be explained by this second factor. However, in order to reduce aerosol generating procedures that may propagate the coronavirus, relevant medical professionals may omit the routine use of nasoendoscopy, serving as another barrier to prompt diagnosis. Other cancer types also saw reduced diagnostic procedures being done during this period [5].

The impact of delayed initial management of curable cancer varies across studies for various cancer types. The United Kingdom recently projected that delays in diagnosis and treatment may increase mortality from breast, colorectal, and lung cancers by as much as 9.6%, 16.6%, and 5.3%, respectively, after 5 years [6]. A delay in diagnosis and treatment may allow cancer to progress to a more advanced stage, thereby, increasing poor outcomes. In our institution, there were more stage 3-4A patients (65%) at diagnosis compared to Stage 1–2 but this was similarly observed in 2017 and 2019. Nevertheless, a multicentre study in England and Northern Ireland estimated effects of COVID-19 on mortality in their study population as well as in the US using the SEER program data. Their model estimated 6,270 excess deaths at 1 year in England and 33,890 excess deaths in the US [11]. Meanwhile a French nationwide study estimated an excess mortality due to cancer of 1000–6000 patients in coming years [1]. Healthcare policies will need to factor in the long term impact of the pandemic on cancer patients and ensure the continued safe running of cancer services.

In our study, despite the reduction in numbers of newly diagnosed NPC, there was no delay from biopsy to the first radiation oncology visit and from biopsy to the first day of treatment in 2020 compared to the preceding years. All the patients treated in our centre also received the standard NPC treatment of either full course of RT alone, concurrent chemoradiation or induction chemotherapy followed by concurrent chemoradiation. Radiation therapy delivered to all patients was the standard fractionation of 70 Grays in 33 fractions over 6.5 weeks, with no shortening of RT regimens to reduce resource utilization. In a separate study in our cancer institution, the authors found no significant drop in the active treatment instituted in patients (as reflected by chemotherapy chair utilization, radiotherapy visits and cancer related surgery) compared to one year prior to the pandemic [7]. This is in contrast to published reports of delays in cancer care elsewhere [8]. A comprehensive systemic review of 62 studies reported significant treatment delay though of varying degrees across the studies included [10]. This was especially seen in radiotherapy where there was a median of 8–45 days delay and 10.3%—80% of centres reported interruption of radiotherapy. 97.4% centres reported a change in radiotherapy schemes to treatments utilizing reduced number of fractions thus shortening the overall radiotherapy course per patient and 100% of centres reported a reduction in the number of radiotherapy sessions. However, the review may not be completely representative as it included higher number of studies from severely affected countries in the first wave of the COVID-19 pandemic such as Italy, USA and China. Of note, Italy and the UK for example, were severely affected by the COVID-19 pandemic, seeing several months of lockdown and an overwhelmed healthcare system with 241,000–286,000 confirmed COVID-19 infections and 35,000–40,600 deaths by June 2020 [3]. Low-income countries such as Africa and India were not included, and neither were less severely impacted countries such as Taiwan and Singapore. In Singapore, the climate was different to these countries. For a large part, the infections mainly clustered in foreign worker dormitories, which were quickly ring-fenced. As the COVID-19 infections mainly occurred in this young and healthy population, the large majority of patients did not suffer from severe illness requiring oxygen or intensive care, thus the health system was fortunately able to avoid being overtaxed. At the end of June 2020, Singapore only had 26 deaths from COVID-19, and at the moment this number stands at 36 (June 2021). Patients’ confidence in their safety within the public areas including hospitals and the country’s less overwhelmed healthcare system may explain the ability of our institution to provide continued cancer services without significant delays in treatment compared to pre-COVID times.

Within the hospital inpatient setting, all patients were required to undergo a pre-admission COVID polymerase chain reaction (PCR) swab before admission, even if asymptomatic. In addition, inpatients undergo a weekly COVID-19 PCR swab during their stay. During the early days of the pandemic, specialised dedicated COVID-19 wards were set aside within the hospital. As vaccination rates increased in Singapore and the country moved towards living with COVID-19, this was stepped down to subspeciality – run COVID cohort wards within each subspeciality department to reduce the strain on beds and manpower.

In addition, the hospital pioneered “virtual” COVID wards where oncology and other high-risk patients who are stable and fulfilling certain criteria are allowed to recover at home. These patients receive regular video consultations over online video conferencing platforms with medical staff, as well as regular self-monitoring of vital signs which are submitted by the patient over online platforms. Medication is home delivered to the patient and they have direct hotlines to call in case of emergencies.

Within the outpatient oncology setting, especially for radiation therapy which requires daily visits for RT, special time slots at the end of the day and treatment rooms were set aside for COVID positive patients to minimise contact with other patients. Staff would attend to these patients in full personal protective equipment (PPE) and the treatment unit would be fully cleaned and sterilised in between each COVID patient. With the introduction of home recovery and the “virtual” COVID wards, patients recovering at home had special transport services arranged for them bringing them for RT and back home immediately. As much as possible, the aim was to achieve < 3 days of treatment disruption for patients undergoing radical treatment, with slightly longer breaks permissible for more indolent cancers or palliative cancers.

These findings highlight that with effective response to the pandemic such as border control, contact tracing and quarantine measures, as well as resource preparedness such as the dedicated National Centre of Infectious Disease, community centres for housing COVID-19 patients and appropriate reallocation of healthcare resources, we may be able to limit disruptions to the care of oncology patients. Our institution was also quick to adopt policies to reduce the risk of COVID-19 exposure whilst ensuring patients obtain appropriate care [13].

Limitations to this study include the retrospective design and that only once cancer type was investigated. While this was a single centre study, our centre sees 60 to 70% on average of cases in the country; as Singapore only has 3 public hospitals offering radiation therapy, serving 90% of the population. Further multicentre exploration into other cancer types will be beneficial to judge the overall impact of COVID-19 on cancer care in the country. Additionally, we only captured patients who underwent treatment. There may be patients who were not referred to our department or did not attend their initial consults if they decline treatment after their diagnosis. The strengths of our study were that we analyzed data from the preceding 3 years for comparison. The sample size of this study was small but it was representative of real world numbers of NPC presentation to our department. Also, the choice of a single cancer subsite, NPC, for analysis, while providing limited data, however allows us to have a clear direct estimate of the impact of COVID-19 on service provision in our centre. Most cancer treatment is generally complex with the involvement of multiple healthcare providers and multiple different possible treatment regiments. In a retrospective study such as this, it may be difficult to account for delays at the various time points in an inhomogeneous group requiring different investigations and the involvement of different healthcare providers. This explains our decision to focus only on NPC, which, as previously mentioned, has clear algorithms for treatment with the radiation oncologist playing a central coordinating role. We hope that this study provides a snapshot into the short term impact of the pandemic on the adequacy of cancer treatment provision in Singapore. While our 3-year survival numbers across the cohorts does show a trend towards slightly worse outcomes in 2020, the small numbers make it difficult to draw conclusions on this. Longer term follow up with a larger cohort across the whole pandemic will be useful to see if the pandemic influences the survival outcomes for these patients. Another meaningful parameter to look at will be if we see any catch-up effect in terms of patient numbers once the impact of the COVID-19 pandemic is reduced, with the rolling out of vaccines and other public health measures to reduce transmission.

We provide the first look into the impact of the COVID-19 pandemic on the presentation and management of NPC within a local institution to better inform about cancer care within this region and to guide policy change. At this time, we are seeing a resurgence of COVID-19 in the Asia–Pacific region. In Singapore itself, a second wave with community cases has resulted in the re-implementation of restrictions as part of ‘heightened measures’ to cull further spread. The pandemic may be far from over and the resulting disruptions with its afterwaves present significant challenges in the rebuilding of socio-economic and healthcare systems. We recommend a heightened awareness of the dangers of delaying cancer presentation and care when planning for healthcare policies and resource allocation and at the same time, encourage patient’s confidence in their ability to seek care.

## Data Availability

All data generated or analysed during this study are included in this published article.

## Abbreviations

DORSCON: Disease outbreak response system condition, a colour coded framework used in Singapore to represent current disease outbreak conditions
NPC: Nasopharyngeal carcinoma
ENT: Ear, Nose and Throat specialists
EMR: Electronic medical records
MRI: Magnetic resonance imaging
PET: Positron emission tomography
VMAT: Volumetric arc radiation therapy
IMRT: Intensity modulated radiation therapy
RT: Radiation therapy
